# Use of Serogroup B Meningococcal Vaccines in Persons Aged ≥10 Years at Increased Risk for Serogroup B Meningococcal Disease: Recommendations of the Advisory Committee on Immunization Practices, 2015

**Published:** 2015-06-12

**Authors:** Temitope Folaranmi, Lorry Rubin, Stacey W. Martin, Manisha Patel, Jessica R. MacNeil

**Affiliations:** 1 Epidemic Intelligence Service, CDC; 2 Advisory Committee on Immunization Practices Meningococcal Vaccines Work Group, Steven and Alexandra Cohen Children’s Medical Center of New York, New Hyde Park, New York and Hofstra North Shore-LIJ School of Medicine, Hempstead, NY; 3 Meningitis and Vaccine Preventable Diseases Branch, Division of Bacterial Diseases, National Center for Immunization and Respiratory Diseases, CDC

In October 2014, the Food and Drug Administration (FDA) licensed the first serogroup B meningococcal (MenB) vaccine (MenB-FHbp [Trumenba, Wyeth Pharmaceuticals, Inc.]) as a 3-dose series. In January 2015, FDA licensed a second MenB vaccine (MenB-4C [Bexsero, Novartis Vaccines]) as a 2-dose series. Both vaccines were approved for use in persons aged 10–25 years. Following outbreaks of serogroup B meningococcal disease on two college campuses in 2013, both MenB vaccines were granted Breakthrough Therapy designations, which expedites drug development and review by FDA, and were licensed based on accelerated approval regulations (1). On February 26, 2015, the Advisory Committee on Immunization Practices (ACIP) recommended use of MenB vaccines among certain groups of persons aged ≥10 years who are at increased risk for serogroup B meningococcal disease. This report summarizes information on MenB administration and provides recommendations and guidance for use of these vaccines among persons aged ≥10 years in certain groups who are at increased risk for serogroup B meningococcal disease, and reviews the evidence considered by ACIP to make these recommendations. Recommendations for broader use of MenB vaccines in adolescents and college students will be considered separately by ACIP.

## Methods

The ACIP Meningococcal Vaccines Work Group reviewed safety and immunogenicity data from seven clinical trials of MenB-4C (2–7) (Novartis, unpublished data) and nine clinical trials of MenB-FHbp (8–13) (Pfizer, unpublished data) during its monthly teleconferences. The Work Group also evaluated published peer-reviewed literature and unpublished data on meningococcal disease epidemiology in the United States. A summary of the data reviewed and Work Group discussions was presented to ACIP, and recommendations for use of MenB vaccines among persons aged ≥10 years at increased risk for serogroup B meningococcal disease were approved by ACIP at its February 26, 2015, meeting (meeting minutes are available at http://www.cdc.gov/vaccines/acip/meetings/meetings-info.html).

The type and quality of evidence supporting the use of MenB vaccines in persons aged ≥10 years at increased risk for serogroup B meningococcal disease was evaluated using the Grading of Recommendations, Assessment, Development, and Evaluation (GRADE) framework (14), and determined to be type 2 (moderate level of evidence) for use in outbreak settings, and type 3 (low level of evidence) for use in persons at increased risk for serogroup B meningococcal disease. The recommendation was designated Category A (recommended for all persons in an age-based or risk-factor–based group) (15).

## Persons at Increased Risk for Meningococcal Disease

Persons who have persistent deficiencies (e.g., genetic deficiencies) in the complement pathway (e.g., C3, properdin, Factor D, Factor H, or C5–C9) have up to a 10,000-fold increased risk for meningococcal disease and can experience recurrent disease (16,17). Persons receiving eculizumab (Soliris, Alexion Pharmaceuticals) for treatment of atypical hemolytic uremic syndrome or paroxysmal nocturnal hemoglobinuria also are at increased risk because the drug binds to C5 and inhibits the terminal complement pathway (information available at http://soliris.net/sites/default/files/assets/soliris_pi.pdf). Similarly, persons with functional or anatomic asplenia (including persons with sickle cell disease) appear to be at increased risk for meningococcal disease, and have a higher mortality rate (40%–70%) from the disease than healthy populations (18). Among microbiologists who routinely work with *Neisseria meningitidis* isolates, the attack rate of laboratory-acquired meningococcal infection has been estimated at 13 per 100,000 persons, which is many fold higher than the rate for adults in the general population (19). In the United States, 97%–98% of all cases of meningococcal disease are sporadic; however, outbreaks continue to occur. Recently, outbreaks of serogroup B meningococcal disease have been reported from several college campuses. Data from four college campus outbreaks (March 2013–May 2015) showed a 200 to 1,400–fold increase in risk for meningococcal disease among students at these colleges during the outbreak period compared with the general population in this age group (Division of Bacterial Diseases, National Center for Immunization and Respiratory Diseases, CDC, unpublished data, 2015).

## MenB Vaccine Immunogenicity and Safety

Because of the low incidence of serogroup B meningococcal disease, vaccine efficacy estimates were based on demonstration of immune response, as measured by serum bactericidal activity using human complement (hSBA), against a small number of serogroup B strains. In studies supporting U.S. licensure, immunogenicity was assessed by the proportion of subjects who achieved a ≥4-fold increase in hSBA titer for each of the strains tested, and the proportion of subjects who achieved a titer greater than or equal to the lower limit of quantification of the assay for all strains (composite response) (20,21). The lower limit of quantification was defined as the lowest amount of the antibody in a sample that can be reliably quantified.

## MenB-4C Vaccine

MenB-4C consists of three recombinant proteins (Neisserial adhesin A [NadA], factor H binding protein [FHbp] fusion protein, and Neisserial Heparin Binding Antigen [NHBA] fusion protein), and outer membrane vesicles (OMVs) containing outer membrane protein PorA serosubtype P1.4. MenB-4C is licensed as a 2-dose series, with doses administered at least 1 month apart, although in some studies, MenB-4C doses were administered up to 6 months apart.

In persons aged ≥10 years, safety and immunogenicity of MenB-4C were evaluated in seven clinical trials: six randomized controlled trials and one immunogenicity extension study (2–7) (Novartis, unpublished data). In one randomized controlled trial conducted in the United Kingdom, a subset of enrolled subjects (university students aged 18–24 years) received 2 doses of MenB-4C vaccine 1 month apart. One month following the second dose, 88% (95% confidence interval [CI] = 82%–93%) of subjects had a composite hSBA response to all three test strains; 66% (CI = 58%–72%) of the subjects had a composite hSBA response to all three test strains at 11 months after the second dose (20). In a randomized controlled trial conducted in Australia and Canada, persons aged 11–17 years received 2 doses of MenB-4C 1 month apart. One month following the second dose, 63% (CI = 57%–68%) of subjects had a composite hSBA response to all three test strains (20).

In an open-label study conducted in Germany and Italy, antibody responses to MenB-4C were assessed in laboratory workers aged 18–50 years who were routinely exposed to *Neisseria meningitidis.* Among the subjects, 83% (CI = 69%–92%) achieved an hSBA titer ≥1:8 against at least one of the three test strains 1 month after the second dose of MenB-4C (3).

In three clinical trials for which a control group was available, serious adverse events were assessed in 2,716 participants who received at least 1 dose of MenB-4C and for whom safety data were collected through 6 months postvaccination (2,4,6). Five serious adverse events were considered by the study investigator to be related (or possibly related) to the vaccine.* Rates of serious adverse events were similar in the vaccine and the control groups. In addition, information about serious adverse events was collected during three prelicensure vaccination campaigns in response to three outbreaks of serogroup B meningococcal disease (at two universities in the United States and in one region in Canada). A total of 59,091 participants in the vaccination campaigns received at least 1 dose of MenB-4C. Three serious adverse events were considered by the study investigator to be related (or possibly related) to the vaccine†; all resolved with no sequelae (CDC and Novartis, unpublished data). No deaths were considered to be related to MenB-4C in the clinical trials or campaigns. The most common solicited adverse reactions observed in the 7 days after receipt of MenB-4C in the clinical trials were pain at the injection site, myalgia, erythema, fatigue, headache, induration, nausea, and arthralgia (9,20).

Safety and immunogenicity data regarding MenB-4C when co-administered with vaccines routinely administered to U.S. adolescents are not available.

## MenB-FHbp Vaccine

MenB-FHbp consists of two purified recombinant FHbp antigens. One antigen from each FHbp subfamily (A and B) is included in the vaccine. MenB-FHbp is licensed as a 3-dose series, with the second and third doses administered 2 and 6 months after the first dose.

Safety and immunogenicity of MenB-FHbp in persons aged ≥10 years were evaluated in nine clinical trials: six randomized controlled trials and three open label studies (8–13) (Pfizer, unpublished data). In a multicenter trial conducted in the United States, persons aged 11–17 years were randomly assigned to one of three groups. Group 1 received MenB-FHbp and quadrivalent human papillomavirus vaccine (4vHPV, [Gardasil Merck and Co.]), group 2 received MenB-FHbp and saline, and group 3 received 4vHPV and saline.

One month following the third dose, 81% (CI = 78.0%–83.7%) of subjects in group 1 and 83.9% (CI = 81.1%–86.4%) of subjects in group 2 had a composite hSBA response to all four test strains (13,21). After the second of 3 doses, approximately 50% of the subjects in each study group had a composite hSBA response to all test strains. In studies conducted among European persons aged 11–18 years, the hSBA responses in subjects who received MenB-FHbp according to the same schedule were similar to hSBA antibody responses in subjects in the U.S. study (9,21).

In one open label study, immunogenicity was assessed among a small number of meningococcal laboratory workers who received the vaccine. Among the subjects, 50% achieved a titer greater than or equal to the lower limit of quantification to all four test strains (Pfizer, unpublished data).

Concomitant administration of MenB-FHbp with vaccines routinely administered to U.S. adolescents has been evaluated in three trials. Subjects received MenB-FHbp co-administered with 4vHPV (Gardasil, Merck and Co.), MenACWY (Menactra, Sanofi Pasteur), Tdap (Adacel, Sanofi Pasteur) or dTaP/IPV (Repevax, Sanofi Pasteur) vaccines. Except for the antibody response to HPV type 18, no immunologic interference was observed for serogroup B or concomitant vaccine antigens (HPV types 6, 11, 16, MenACWY, tetanus, diphtheria, pertussis and IPV antigens) when MenB-FHbp was administered concomitantly (11,13) (Pfizer, unpublished data). For HPV type 18, noninferiority criteria (lower bound of the CI of the geometric mean titer ratio >0.67) were not met for the geometric mean titer ratio at 1 month after the third 4vHPV vaccination (lower bound of the CI for the geometric mean titer ratio was 0.62); however, ≥99% of subjects achieved seroconversion for all four HPV antigens.

In four clinical trials (9,11–13) a total of 2,557 subjects received at least 1 dose of MenB-FHbp; four subjects reported seven serious adverse events that were considered by the study investigator to be related (or possibly related) to the vaccine.§ All vaccine-related serious adverse events resolved without sequelae. No increased risk for any specific serious adverse event considered to be clinically significant was identified in any of the studies. No deaths were considered to be related to MenB-FHbp. The most common solicited adverse reactions observed in the 7 days after receipt of MenB-FHbp in the clinical trials were pain at the injection site, fatigue, headache, myalgia, and chills (21).

## Rationale for Recommendations

Certain groups of persons known to be at increased risk for meningococcal disease are recommended to be routinely vaccinated with a quadrivalent meningococcal conjugate vaccine (MenACWY), which protects against serogroups A, C, W, and Y (16). Many of these groups are also at increased risk for serogroup B meningococcal disease. Available immunogenicity and safety data support the use of MenB vaccines in groups at increased risk for serogroup B meningococcal disease.

Both MenB vaccines are approved for use in persons aged 10–25 years; however, because there are no theoretical differences in safety for persons aged >25 years compared with those aged 10–25 years, ACIP supported routine use of MenB vaccines in persons aged ≥10 years who are at increased risk for serogroup B meningococcal disease. These recommendations do not apply to children aged <10 years.

## Recommendations

Certain persons aged ≥10 years who are at increased risk for meningococcal disease should receive MenB vaccine. These persons include:

- Persons with persistent complement component deficiencies.¶
- Persons with anatomic or functional asplenia.**
- Microbiologists routinely exposed to isolates of *Neisseria meningitidis*.
- Persons identified as at increased risk because of a serogroup B meningococcal disease outbreak.

Certain other groups are included in the MenACWY recommendations for persons at increased risk, but are not included in this recommendation. MenB vaccines are not licensed for children aged <10 years and are not currently recommended for children aged 2 months–9 years who are at increased risk for serogroup B meningococcal disease. MenB vaccine is not recommended for persons who travel to or reside in countries where meningococcal disease is hyperendemic or epidemic because the risk for meningococcal disease in these countries generally is not caused by serogroup B. The vaccine is not currently recommended for routine use in first-year college students living in residence halls, military recruits, or all adolescents. Recommendations for broader use of MenB vaccines in adolescents and college students will be considered separately by the ACIP.

What is currently recommended?

The Advisory Committee on Immunization Practices recommends routine vaccination with quadrivalent meningococcal conjugate vaccine (MenACWY) of certain groups of persons at increased risk for meningococcal disease: persons who have persistent complement component deficiencies; persons who have anatomic or functional asplenia; microbiologists who routinely are exposed to isolates of *Neisseria meningitidis;* persons identified to be at increased risk because of a meningococcal disease outbreak attributable to serogroup A, C, W, or Y; military recruits; first-year college students living in residence halls; and persons who travel to or reside in areas in which meningococcal disease is hyperendemic or epidemic.

Why are the recommendations being modified now?

Two serogroup B meningococcal (MenB) vaccines were recently licensed by the Food and Drug Administration and approved for use in persons aged 10–25 years. The evidence supporting the use of MenB vaccines in persons at increased risk for serogroup B meningococcal disease was evaluated using the Grading of Recommendations, Assessment, Development, and Evaluation framework and determined to be type 2 (moderate level of evidence) for use in outbreak settings and type 3 (low level of evidence) for use in persons at increased risk for serogroup B meningococcal disease. The recommendation was designated as Category A (recommended for all persons in an age- or risk-factor–based group).

What are the new recommendations?

Certain persons aged ≥10 years at increased risk for meningococcal disease should receive MenB vaccine. These persons include those with persistent complement component deficiencies; persons with anatomic or functional asplenia; microbiologists routinely exposed to isolates of *Neisseria meningitidis;* and persons identified to be at increased risk because of a serogroup B meningococcal disease outbreak.

MenB vaccine should be administered as either a 2-dose series of MenB-4C or a 3-dose series of MenB-FHbp. The same vaccine product should be used for all doses. Based on available data and expert opinion, MenB-4C or MenB-FHbp may be administered concomitantly with MenACWY vaccines, but at a different anatomic site, if feasible.

## Precautions and Contraindications

Before administering MenB vaccines, providers should consult the package insert for precautions, warnings, and contraindications (20,21). Adverse events occurring after administration of any vaccine should be reported to the Vaccine Adverse Event Reporting System (VAERS). Reports can be submitted to VAERS online, by fax, or by mail. Additional information about VAERS is available by telephone (1–800–822–7967) or online (http://vaers.hhs.gov).

## Footnotes

* The administration of the investigational vaccine and a serious adverse event were considered reasonably related in time and the serious adverse event could not be explained by causes other than exposure to the investigational vaccine. The reported serious adverse events included tremor (one), dyspnea (one), acute thyroiditis (one), and juvenile arthritis (two).

† The reported serious adverse events included rhabdomyolysis (one), anaphylaxis (one) and fever (one).

§ The reported serious adverse events included pyrexia (one), vomiting (one), vertigo (one), chills (one), headache (one), anaphylaxis (one) and neutropenia (one).

¶ Including inherited or chronic deficiencies in C3, C5-9, properdin, factor D, factor H, or who are taking eculizumab (Soliris).

** Including sickle cell disease.
